# Pelvic Exenteration for Recurrent Endometrial Cancer: A 15-Year Monocentric Retrospective Study

**DOI:** 10.3390/cancers15194725

**Published:** 2023-09-26

**Authors:** Nando Fix, Sabrina Classen-von Spee, Saher Baransi, Verónica Luengas-Würzinger, Friederike Rawert, Ruth Lippert, Peter Mallmann, Björn Lampe

**Affiliations:** 1 Department of Gynecology and Obstetrics, Florence-Nightingale-Hospital, Kreuzbergstraße 79, 40489 Düsseldorf, Germany; classen@kaiserswerther-diakonie.de (S.C.-v.S.); baransi@kaiserswerther-diakonie.de (S.B.); luengas-wuerzinger@kaiserswerther-diakonie.de (V.L.-W.); rawert@kaiserswerther-diakonie.de (F.R.); lampe@kaiserswerther-diakonie.de (B.L.); 2 Department of Pathology, Evangelisches Krankenhaus Oberhausen, Virchowstraße 20, 46047 Oberhausen, Germany; ruth.lippert@eko.de; 3 Department of Obstetrics and Gynecology, University of Cologne, Kerpener Str. 62, 50937 Cologne, Germany; peter.mallmann@uk-koeln.de

**Keywords:** recurrent endometrial cancer, pelvic exenteration, multivisceral surgery

## Abstract

**Simple Summary:**

The incidence of endometrial cancer is continuously rising within the last few decades. The diverse patient population impairs standardized procedures and asks for individualized treatment options. If patients already received or failed to respond to radio- or chemotherapy, secondary surgical procedures such as pelvic exenteration might be the only curative option. The heterogeneity of the published data is a big challenge for an interpretation of long-term survival after pelvic exenteration. This study retrospectively analyzed data of a homogenous patient population receiving pelvic exenteration. When complete cytoreduction was achieved, a substantial overall survival was measured. Declining morbidity and mortality rates support pelvic exenteration as a valid treatment option for carefully chosen patients with recurrent endometrial cancer.

**Abstract:**

Treatment options for recurrent endometrial adenocarcinoma are limited. In those cases, secondary surgical procedures such as pelvic exenteration form the only possible curative approach. The aim of this study was analyzing the outcomes of patients who underwent pelvic exenteration during the treatment of recurrent endometrial cancer intending to identify prognostic factors. More than 300 pelvic exenterations were performed. Fifteen patients were selected that received pelvic exenteration for recurrent endometrial adenocarcinoma. Data regarding patient characteristics, indication for surgery, complete cytoreduction, tumor grading and p53- and L1CAM-expression were collected and statistically evaluated. Univariate Cox regression was performed to identify predictive factors for long-term survival. The mean survival after pelvic exenteration for the whole patient population was 22.7 months, with the longest survival reaching up to 69 months. Overall survival was significantly longer for patients with a curative treatment intention (*p* = 0.015) and for patients with a well or moderately differentiated adenocarcinoma (*p* = 0.014). Complete cytoreduction seemed favorable with a mean survival of 32 months in contrast to 10 months when complete cytoreduction was not achieved. Pelvic exenteration is a possible treatment option for a selected group of patients resulting in a mean survival of nearly two years, offering a substantial prognostic improvement.

## 1. Introduction

Endometrial cancer (EC) is the gynecologic malignancy with the highest incidence in postmenopausal women, and its diagnosis has increased 130% within the last 30 years [1,2]. Unlike other gynecologic malignancies, EC is commonly diagnosed in an early stage due to the presence of symptoms (usually bleeding), which results in long-term survival for most patients. Approximately 15–20% of EC patients have aggressive tumor characteristics indicating a higher risk for distant metastasis. In those cases, additional abdominal lymphonodectomy is performed and followed by radiotherapy or even chemoradiotherapy [1]. Even though tumor-specific pharmacological treatments focused on tumor biology and patient characteristics are getting more relevant, the first step in successfully treating EC is to achieve a complete cytoreduction (R0) [2,3].

Although EC recurrence rates are low, they usually present with a disseminate abdominal disease. Treatment options are limited due to the fact that most patients already had a combination of surgery and radio-/chemotherapy during their initial treatment [4]. A small subset of these patients will develop isolated recurrence within the pelvis, making them possible candidates for surgical treatment. Especially if patients already received or failed to respond to radiotherapy, secondary surgical procedures might be the only curative option, usually consisting of an extensive (often multivisceral) approach.

Pelvic exenteration (PE) is one of the most radical surgical approaches. It was first described in 1948 as a treatment for advanced pelvic malignancies [5]. Since then, the surgical techniques and perioperative management of patients have improved, resulting in lower mortality and morbidity, which has broadened the indication for this surgery. Previously inoperable patients or those suffering tremendously of tumor-related symptoms are now considered suitable for a possibly curative approach [6].

Even though PE is most commonly applied for patients with cervical cancer, there are also indications in the treatment of EC [7]. As obesity is one of the strongest risk factors for EC, patients usually possess a high body mass index, an advanced age and are often multimorbid. All those characteristics turn them into unsuitable candidates for multivisceral surgery, resulting in low indication rates for PE [2,8]. Until now, only a few published articles have evaluated the outcomes of PE in the treatment of recurrent EC. This leads up to the question if PE should be considered as a valid therapeutic option in the treatment of recurrent EC.

The discovery of four genomic EC subtypes by the Cancer Genome Atlas (TCGA) endometrial collaborative project in 2013 and the subsequent validation of the molecular based Proactive Molecular Risk Classifier for Endometrial Cancer (ProMise) in 2018 led to a risk classification that helps to determine therapeutic strategies (e.g., the need for adjuvant therapy) [9,10]. The four molecular subtypes are the following: *p53* wildtype, *p53* abnormal, *POLE* mutated and mismatch repair deficient. The worst prognosis of all molecular subtypes is related to alterations, either overexpression or missense, of the tumor suppressor *TP53* and includes 8–24% of ECs [10,11,12]. Mutations of p53 lead to an intermediate or high risk classification. The polymerase epsilon (POLE) is a DNA replicate that has a proofreading domain associated with low mutation rates in DNA replication. Mutations appear in approximately 10% of EC, have an excellent prognostic influence and result in a low risk classification. Loss of one or more mismatch repair proteins (mismatch repair deficient) is detected in 23–36% of EC and causes microsatellite instability, which can be found in many cancer types [12]. In addition to the ProMise classification, the L1 cell adhesion molecule (L1CAM) further stratifies the EC risk classification. L1CAM is overexpressed in 7–18% of EC and associated with distant recurrence, overall survival and p53 expression [12,13,14].

The aim of this study was to analyze the outcomes of patients who underwent PE during the treatment of recurrent EC aiming to identify positive prognostic factors. Therefore, data from our oncological center was analyzed and correlated with the current literature.

## 2. Materials and Methods

### 2.1. Study Design and Population

A retrospective data analysis was performed for all patients with recurrent endometrial cancer that received a pelvic exenteration at the Florence Nightingale Hospital between January 2007 and December 2022. This hospital is accredited by the European Society of Gynecological Oncology (ESGO) as a center of training in gynecological oncology and has performed more than 300 pelvic exenterations in the last 16 years. Patient data were retrieved from clinical reports and collected in a retrospective database.

**Scheme 1:**
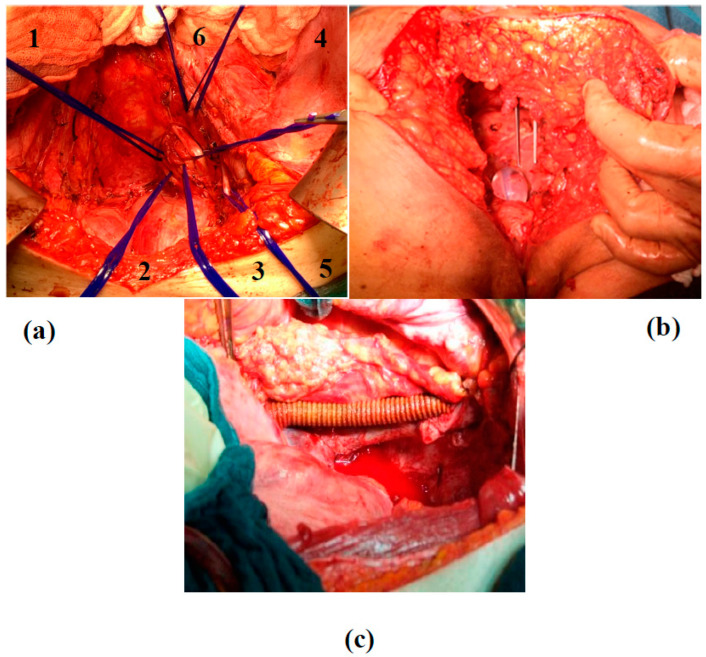
Spectrum of pelvic exenteration; (**a**) Complete pelvic exenteration, including removal of the vagina, uterus, ovaries, fallopian tubes, bladder and the rectum. Resection of the left pelvic sidewall with preparation of the plexus sacralis (1–4), nervus obturatorius (5) and nervus femoralis (6); (**b**) Resection of the os sacrum during complete pelvic exenteration; (**c**) Resection of the left external iliac artery with implantation of a prosthesis (main surgeon: B. Lampe).

Recurrent disease was defined as detection of locoregional relapse or distant metastasis. Preoperative vaginal biopsy or intraoperative frozen section diagnosis was used to histologically prove disease recurrence. Pelvic exenteration was defined as resection of the vagina, uterus, ovaries, fallopian tubes and the bladder (anterior exenteration), or the rectum (posterior exenteration), or a combination of both (total exenteration). Surgeries were mainly driven by the aim of complete cytoreduction requiring adapted procedures that went beyond the given definitions. PE included a broad spectrum of surgical approaches such as dissection of the lateral pelvic structures, defined by the Triangle of Marcille, and bone structures (Scheme 1). All patients with histologically proven recurrent type I endometrial cancer that received PE were included. All types of pelvic exenteration (anterior, posterior and total) as well as all histological subtypes of type I endometrial cancers, according to the World Health Organization (WHO) classification, were included. Patients with type II carcinomas (serous, clear cell), with type I carcinomas treated with systematic therapy only, sarcomas or carcinosarcomas were excluded.

Patient characteristics have been analyzed by calculating the mean with interquartile range. All patients received a chest and abdominal computed tomography (CT) scan, and tumor size was analyzed by magnetic resonance imaging (MRI). Initially, all patients were discussed in a tumor board consisting of a gynecologist, medical oncologist, radiotherapist, pathologist, surgical oncologist and radiologist. The primary endpoint of the analysis was overall survival (OS), defined as duration from the date of surgery to the death of a patient or date of last contact. A univariate Cox regression was performed to identify variables that influence clinical outcome. The dependent variable was defined as the survival time after pelvic exenteration in months. Independent variables were as follows: indication for surgery (palliative vs. curative), tumor grading (G1/2 vs. G3), p53-mutation status, L1CAM-status and complete cytoreduction (R1 vs. R0). A loco regional recurrence where a complete gross resection seemed achievable, without the presence of distant metastasis, received a curative indication for surgery. A palliative surgery indication was defined as preoperative detection of distant metastasis or impossibility of achieving locoregional complete tumor resection (e.g., due to nerval infiltration). In those cases, pelvic exenteration was performed with the aim to improve quality of life and to relieve tumor-related symptoms (e.g., fistula formation or recurrent bleeding). Tumor grading was performed according to the recommendations of the International Federation of Gynecology and Obstetrics (FIGO) and the WHO and categorized as well differentiated (G1), moderately differentiated (G2) and poorly differentiated (G3). For p53- and L1CAM analysis, blank 4 µm sections were cut from corresponding formalin-fixed, paraffin-embedded EC tissue and stained with p53-antibody (Clone DO-7, Ventana, Oro Volley, AZ, USA); Platform: Ventana BenchMark) and L1CAM-antibody (Clone UJ127, Thermo Fisher, Waltham, MA, USA); Platform DAKO-Autostainer Link 48). L1CAM was considered positive when >10% of the tumor cells expressed L1CAM. Immunostaining for p53 was recorded abnormal when >90% of the nuclei were stained or when no nuclei were stained. Complete macroscopic resection of the tumor, including pathological tumor-free margins, was defined as complete cytoreduction (R0).

Analysis of patient survival was conducted with the Kaplan–Meier estimate. The level of significance was defined at 5%. Statistical analysis was performed using IBM Corp. SPSS (Statistics for Macintosh, Version 28.0.1.1. Armonk, NY, USA).

### 2.2. Literature Review

A comprehensive review of the current literature was performed by two independent researchers utilizing the common databases (Pubmed/MEDLINE, Elsevier and the Cochrane Library). A logic combination of MESH-terms (“pelvic exenteration” and “endometrial neoplasm”) was used to filter the results. All articles from the last 30 years have been included. The literature analysis was conducted following the standards of the Preferred Reporting Items for Systematic Reviews and Meta-Analyses (PRISMA). Articles utilizing a clear methodology that were focused on recurrent endometrial cancer have been included. Exclusion criteria were a lack of statistical analysis of the results, cohort studies without a clear differentiation between tumor entities, case reports, laparoscopic or robotic surgical approaches and articles not written in English. The last date of retrieval was 1 April 2023. No randomized controlled trials or studies utilizing a control group were identified. Therefore, a meta-analysis could not be performed.

## 3. Results

### 3.1. Data Analysis

A total of 326 cases of performed PEs could be retrieved from our database. The most common indication was cervical cancer (39%), followed by vulva/vagina (28%), ovarian (17%), uterine (9%) and other (7%) malignancies. Twenty-five PEs were performed due to EC. After applying the exclusion und inclusion criteria, a total of 15 patients could be retrieved from the database who underwent pelvic exenteration due to recurrent endometrial adenocarcinoma (Table 1). Disease recurrence was detected using CT or MRI and histopathologically proven before or during PE. Mean patient age at date of surgery was 65.9 years, with the youngest patient receiving PE at the age of 54 and the oldest patient with 76 years. All patients had received surgery during their first-line therapy. During their initial treatment, five patients (33.3%) received an adjuvant combination of radio- and chemotherapy, four patients (26.7%) received radiotherapy, three patients (20%) received chemotherapy and three patients (20%) received surgery alone. PE was performed during the first EC recurrence in ten patients (66%). Three patients (20%) underwent PE during the treatment of the second recurrence. One patient (7%) received PE for the third recurrence and one (7%) for the fourth recurrence. One patient had a reported lymphatic- and blood-vessel invasion at the time of surgery. Of the 15 patients included, a total pelvic exenteration (Scheme 1) was performed in nine patients (60%), three patients (20%) received a posterior pelvic exenteration, and three patients (20%) underwent an anterior pelvic exenteration. No patient received intraoperative radiotherapy. Reconstruction of the vagina was not performed as a routine treatment.

**Scheme 2:**
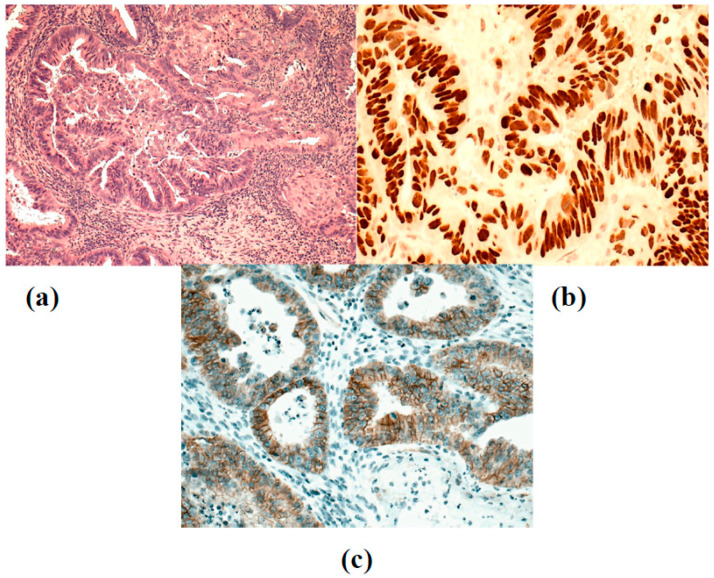
Microscopic images of an endometrioid adenocarcinoma (original magnification ×200); (**a**) hematoxylin and eosin stain of a typical endometrial adenocarcinoma section displaying cells with atypical nuclei and well-formed glands; (**b**) p53-abnormal endometrioid adenocarcinoma with a strong staining of more than 90% of the nuclei; (**c**) L1CAM-positive ECs were identified due to the staining of more >10% of the tumor cells (pathologist: R. Lippert).

All 15 patients had an endometrioid adenocarcinoma of the uterus. An abnormal expression of the tumor suppressor p53 was detected in three patients (20%), and L1CAM overexpression was observed in two patients (13.3%) (Scheme 2). No effects on patient survival were measured with regards to p53- and L1CAM-expression. Univariate analysis of lymphatic- and blood-vessel invasion did not affect patient survival (Table A1). All patient characteristics are summarized in Table 1.

**Figure 1:**
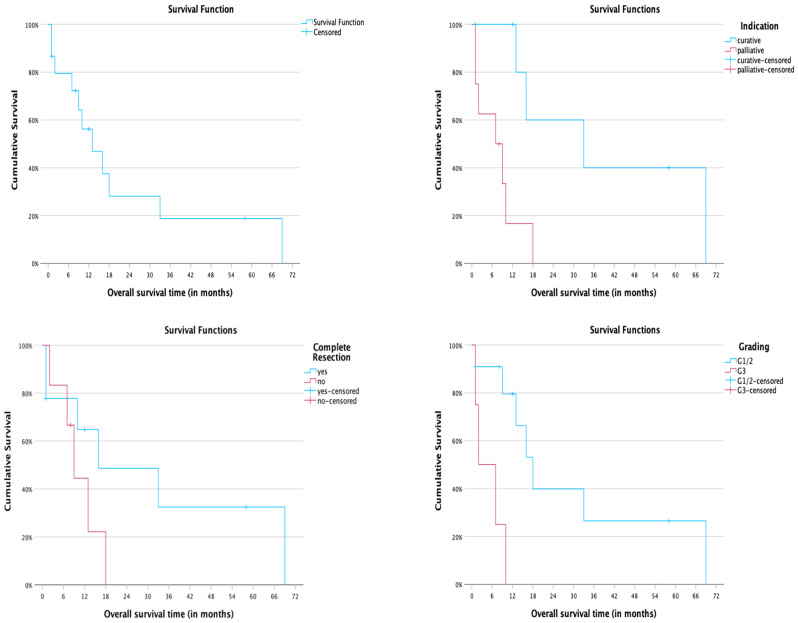
Overall survival of the whole patient cohort in the months after pelvic exenteration. Overall survival analyzed by indication differed significantly (*p* = 0.015). Complete macroscopic and microscopic resection of the tumor was a favorable variable for patient survival (*p* = 0.19). Tumor grading had a prognostic value on patient survival. Patients with a highly differentiated carcinoma had a significant lower overall survival (*p* = 0.014).

The mean survival after pelvic exenteration for the whole patient population was 22.7 months, with the longest survival reaching up to 69 months. Complications requiring surgical, endoscopic or radiological intervention (Clavien–Dindo ≥ 3) were observed in five cases (33.3%). Three palliative patients died one month after PE due to complications. The survival was influenced by the indication for the surgery. Patients with a palliative indication had a mean survival of 7.5 months, which significantly differed from the curative PE indications with a mean survival of 40.0 months (*p* = 0.015). Complete cytoreduction, demonstrated by the pathologist as complete microscopic resection, was achieved in nine patients (60%) and showed a mean survival of 32 months in contrast to ten months when complete cytoreduction was not achieved (*p* = 0.19). Analysis of the tumor grading revealed a mean survival of 30.0 months for patients with a well- or moderately differentiated (G1 or G2) adenocarcinoma and a mean survival of five months for patients with a poorly differentiated (G3) adenocarcinoma (*p* = 0.014) (Figure 1). An overview of the performed univariate analysis of the independent variables is given in Table A1.

### 3.2. Literature Review

**Figure 2:**
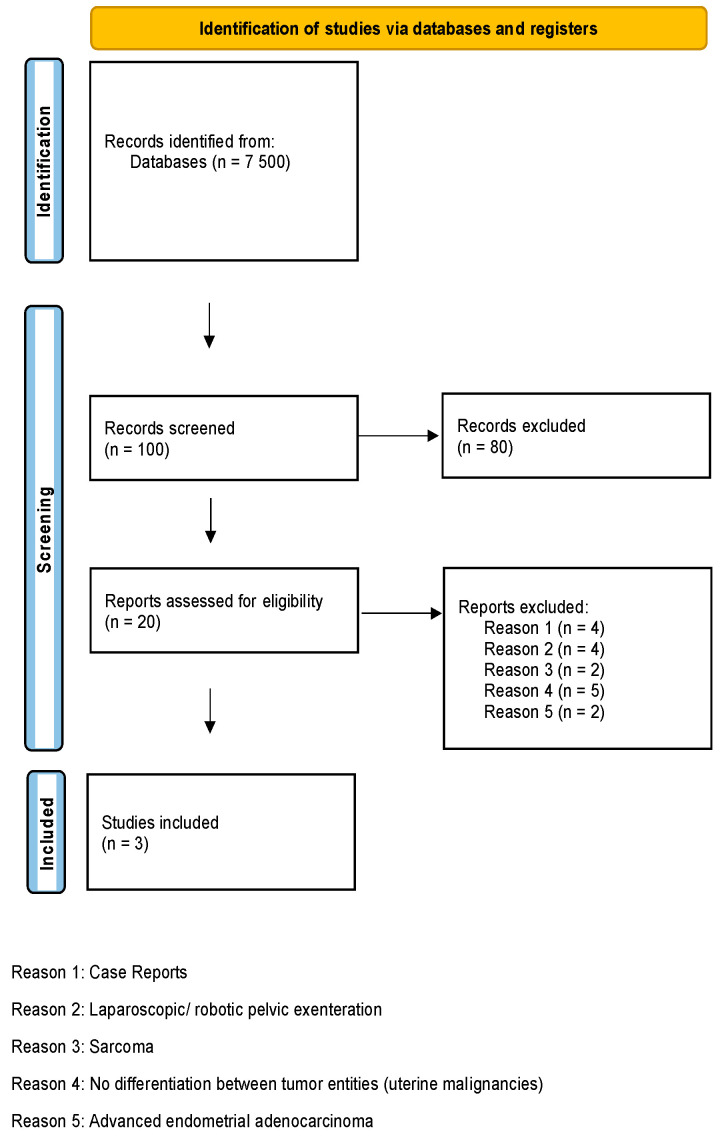
Literature synthesis according to the standards of the Preferred Reporting Items for Systematic Reviews and Meta Analyses (PRISMA).

Initially, approximately 7500 articles were identified searching Pubmed/MEDLINE, Elsevier and the Cochrane Library. Titles and abstracts of 100 articles were screened for relevance after applying MESH-Terms, removing duplicates and narrowing down the publication date to the last 30 years. Twenty articles were retrieved and studied in detail. After an extensive analysis, three articles were identified that matched the inclusion and exclusion criteria (Figure 2).

## 4. Discussion

Patients with recurrent endometrial cancer often face hopeless prognostic situations with only few palliative therapeutic options left. Unlike cervical cancer, which tends to be localized, recurrent EC is known to disseminate within the abdominal cavity or at distant metastatic sites (such as the liver or lungs) [16]. Hence, indications for PE in the treatment of recurrent EC are rare, as central tumor recurrence forms the main indication for a surgical approach. The aim of this study was to evaluate the outcome of patients treated with PE in those situations.

Until today, only a paucity of articles has been published analyzing PE in the treatment of recurrent EC. The three identified articles were published in 1996, 1999 and 2014 and consolidate a total of 85 patients (Table 2). All articles were lacking a control group. Therefore, running a meta-analysis of the published data was not possible. Since the patient data were published in a summarized manner, a pooling of the results could not be realized. Morris et al. (1996) performed an analysis of 20 patients with recurrent endometrial cancer that underwent PE with a curative intention [16]. Their data were collected over 27 years in four different institutions: 90% of the patient population had localized disease, and 60% experienced major postoperative complications (e. g. gastrointestinal or urinary fistulae, paralytic ileus or pelvic abscesses). The calculated 5-year overall survival rate was 56%.

Barakat et al. (1999) reviewed their experience with a total of 44 patients receiving PE with a curative intention for recurrent EC in a single institution over a period of more than 40 years [4]. They reported an overall survival rate after five years of 20% with a postoperative complication rate of 80%.

Chiantera et al. (2014) published the outcome of a total of 21 patients with recurrent EC that underwent PE with a curative intention in four different institutions spread over two countries in a period of 11 years [8]. The resulting complication rate was 43% with a five-year survival rate of 40%.

The conforming conclusion of the mentioned articles was that, despite high complication rates, PE should be considered as a possible treatment option in a highly selected patient population. Including this article, only four studies have been published within the last 27 years evaluating the outcomes of PE in the treatment of recurrent EC, which underlines the need for continued research on this topic.

The heterogeneity of the published data is a big challenge for an interpretation of long-time survival after PE. A separate analysis of different tumor entities is often missing. The lack of a differentiation between advanced and recurrent carcinomas is inhibiting pooling of the published data. Mortality and morbidity are described for PE in general, even though they are not equally distributed among different gynecologic cancers [3,17,18,19,20,21]. The high level of experience and the multidisciplinary involvement required to perform PE results in small case series or case reports [7,22,23,24]. Since PE is occasionally performed during the treatment of recurrent EC, data are often retrieved from different hospitals and countries to gather a sufficient number of patients [8,25].

Due to limited treatment options in recurrent EC, the establishment of a control group is one of the biggest challenges to enable a meta-analysis. After extensive literature research, no randomized controlled trials were identified analyzing the outcomes of PE in the treatment of EC. This underlines the need for differentiated multi-center studies comparing extensive surgery versus non-surgical treatments.

According to the published data, the status of the resection margins is one of the most important prognostic factors for long-time survival after PE, which is supported by the results of this study (HR = 0.41; 95% CI 0.11–1.57) [6]. A meta-analysis of Barlin et al. (2010), including advanced and recurrent EC, suggested complete cytoreduction is associated with a superior overall survival outcome [26]. Moukarzel et al. (2021) analyzed non-exenterative surgical management of recurrent EC in a retrospective study involving 376 patients. A total of 61 patients received secondary surgery, of which, in a majority (75.4%), complete gross resection was achieved. Nevertheless, complete gross resection did not demonstrate a significant survival benefit. Possible explanations given by the authors might be the small comparator group of 24.6% and the small amount of residual disease (<3 cm) in this group [27]. Patients with well- or moderately differentiated EC are benefitting most from PE (*p* = 0.014), as the tumor is not possessing highly aggressive characteristics. L1CAM positivity did not show a statistically relevant influence on patient survival, which may be explained due to the low number of patients reported (13.3%). Kim et al. (2023) recently reported an analysis of 162 patients with EC showing an associated poor prognosis for L1CAM positivity [14].

Nowadays, even distant metastasis is no longer a contra-indication for PE, as the PelvEx collaborative has shown in a study involving 128 patients with rectal cancer that received PE with synchronous liver resection. A 5-year overall survival of 55% was reported with a 30-day mortality of 1.6% [28]. In recent years, the limits of resectability have expended continuously offering curative treatment options for once palliative patients. Tumor infiltration beyond the endopelvic cavity, once a limiting factor for curative surgery, has been tested with complete cytoreduction rates (up to 75%) and acceptable morbidity rates (of 28%) coining the term ‘out-of-the-box surgery’ (Scheme 1) [29,30,31,32]. The reported overall survival for patients with gynecologic malignancies that received complete cytoreduction during ‘out-of-the-box surgery’ was 32 to 60 months [32,33]. Furthermore, if completely removed, pelvic sidewall infiltration does not negatively affect survival [34].

The effect of various comorbidities should also be taken into account as a prognostic factor for patient outcome. Obesity and a high burden of comorbidities are common in EC patients [2]. Di Donato et al. (2021) calculated an age-adjusted Charlson Comorbidity Index (A-CCI) for EC patients receiving surgery. An A-CCI ≥ 3 significantly correlated with more aggressive tumor features, risk of recurrence and death [35]. Although not examined in this study, patient-specific characteristics, as well as tumor-specific characteristics, should influence the therapeutic decisions with the aim of a holistic and individualized approach. The Charlson Comorbidity Index is a promising tool to select patients eligible for PE.

Even though many studies reporting the outcomes of PE are focused on curative approaches, the analysis of palliative PE indications is worth considering. As our data suggest also, palliative patients with distant metastatic disease or inoperable local tumor recurrence might profit from PE and can reach a postoperative survival of up to 18 months. Especially for palliative patients, PE can result in maintenance or improvement of quality of life [36,37]. Several studies have shown that quality of life remains higher for patients that underwent PE compared to those that did not [29]. Even though complications or physical defects might result from PE, patients are able to adapt to those [37]. Patients that did not undergo PE often face a gradual decline in quality of life (e.g., fistula formation, bleeding or urinary and pelvic sepsis) as the disease progresses [29]. Although survival is not prolonged by PE for palliative patients, it can be indicated for a selected patient population with a substantially reduced quality of life.

Improved surgical techniques, peri- and postoperative management and interdisciplinary collaboration have led to a continuous decline in morbidity and mortality [38]. When first described, the initial mortality rate of PE was 23% [5]. A database analysis of 2305 patients that underwent total pelvic exenteration in the United States between 2005 and 2016, including all pelvic malignancies, reported 15% high-grade complications and a mortality rate of 2% [7]. Put into perspective, alternative treatment options such as chemotherapy or immunotherapy are known for severe side-effects. The recently published results of the KEYNOTE-775 trial reported grade 3 or higher adverse events in 90% of the patients that received immunotherapy and in 73% of patients that received chemotherapy during the treatment of advanced or recurrent EC. Median overall survival was 18 months for immunotherapy and 12 months for chemotherapy [39,40]. Our study reported a mean overall survival of 22.7 months after PE (including palliative surgery indications). Additionally, 33.3% of patients had major complications (Clavien–Dindo ≥ 3), which was lower compared to other studies [3,19,33,34,38,41]. If patients are physically stable enough to endure an extensive surgery and gross tumor resection is achievable and not limited by distant metastasis, PE should be considered as a viable treatment option in recurrent EC.

The monocentric and retrospective aspects are the main limitations of this study. The lack of DNA polymerase epsilon (POLE), mismatch repair proteins and the consecutive lack of a molecular risk classification in the data analysis are additional limitations. Most patients (12/15) received PE before ProMise validation in 2018, and molecular classification was not performed on a regular basis. Focusing on one tumor entity with reported data of every patient might establish a meta-analysis, if comparable studies are performed in the future. However, multicentric prospective studies are required to assess the outcome of this ultra-radical surgery.

Women with recurrent EC are often obese, at an advanced age and multimorbid, making them unfit candidates for secondary surgery. PE is reserved for a highly selected patient population, as the procedure requires physically robust patients. Considering tumor biology, tumor-related symptoms, patient wishes and the achievability of a complete cytoreduction, indications for PE should be an individualized decision. Distant metastasis or pelvic side wall infiltration does not impair survival after PE in selected patients. The wide range of survival between 1 and 69 months of the analyzed patients indicates that carefully chosen patients benefit from PE. A “one fits all” approach in the treatment of recurrent EC should be discussed with great caution, as the heterogeneity of the patient population is one of the main limiting factors.

## 5. Conclusions

PE is a possible treatment option for selected patients, resulting in a mean survival of nearly two years and might offer a substantial prognostic improvement for patients with highly limited treatment options.

## Figures and Tables

**Table 1:** From the 25 patients that received a PE due to EC, 15 patients were included. All patients had an endometrioid adenocarcinoma (type I).

Patient	Age at Surgery (Years)	PE	Initial Treatment	Initial FIGO-Stage	Risk Group *	Recurrence	Indication	Grading	Complete Cytoreduction	LVI	BVI	p53	L1CAM	Complications (Clavien–Dindo)
1.	72	Total	S ^1^ + RT ^2^	II	High-intermediate	Second	Curative	G2	R0	L1	V1	M	−	1
2.	63	Total	S ^1^ + RCT ^4^	IB	Intermediate	First	Curative	G2	R1	LX	VX	M	+	1
3.	67	Posterior	S ^1^ + RT ^2^	II	High-intermediate	Second	Curative	G2	R0	LX	VX	WT	−	1
4.	66	Total	S ^1^ + RCT ^4^	Unk.	Unk.	Fourth	Palliative	G2	R0	L0	V0	WT	−	5
5.	66	Total	S ^1^ + RT ^2^	Unk.	Unk.	Second	Curative	G2	R0	L0	V0	WT	−	3b
6.	60	Anterior	S ^1^ + RT ^2^	II	High-intermediate	First	Curative	G2	R0	L0	V0	WT	−	2
7.	61	Anterior	S ^1^ + RCT ^4^	II	High-intermediate	First	Curative	G2	R0	L0	V0	WT	−	1
8.	65	Posterior	S ^1^	IA	Low	First	Palliative	G2	R1	LX	VX	WT	−	1
9.	64	Total	S ^1^ + RCT ^4^	II	High-intermediate	First	Curative	G2	R0	L0	V0	WT	−	1
10.	70	Total	S ^1^ + CT ^3^	IVA	High	First	Palliative	G3	R1	L0	V0	WT	−	5
11.	74	Total	S ^1^ + RCT ^4^	IA	Intermediate	Third	Palliative	G3	R0	LX	VX	WT	−	5
12.	54	Posterior	S ^1^ + CT ^3^	IA	Intermediate	First	Palliative	G3	R1	L0	V0	WT	−	2
13.	67	Total	S ^1^	IA	Low	First	Palliative	G1	R1	L0	V0	WT	−	3b
14.	63	Total	S ^1^	III	High	First	Palliative	G3	R0	L0	V0	M	+	2
15.	76	Anterior	S ^1^ + CT ^3^	III	High	First	Palliative	G2	R1	L0	V0	Unk.	Unk.	2

^1^ Surgery. ^2^ Radiotherapy. ^3^ Chemotherapy. ^4^ Chemoradiotherapy. * Risk groups of endometrial cancer according to the ESGO/ESTRO/ESP guidelines [15]. LVI: lymphatic vessel invasion. BVI: blood vessel invasion. p53 M: mutated. p53 WT: wildtype. L1CAM +: positive. L1CAM −: negative. Unk.: Unknown.

**Table 2:** Characteristics of the extracted articles with a summary of the published results.

Number	Author	Year	Study Design	N	Mean Age (Years)	Complication Rate	Overall Survival Rates at 5 Years
1	Chiantera et al. [8]	2014	retrospective	21	66	43%	40%
2	Barakat et al. [4]	1999	retrospective	44	60	80%	20%
3	Morris et al. [16]	1996	retrospective	20	65	60%	56%

## Data Availability

Data are contained within the article.

## Appendix A

**Table A1:** Results of the univariate analysis with hazard ratio und confidence interval for each independent variable.

Independent Variable	Hazard Ratio
Age at Surgery	HR = 0.98 [95% CI 0.86–1.12]
Indication for Surgery	HR = 7.47 [95% CI 1.47–37.96]
Complete Cytoreduction	HR = 0.41 [95% CI 0.11–1.57]
Tumor Grading	HR = 8.72 [95% CI 1.54–49.42]
Lymphatic Vessel Invasion	HR = 0.04 [95% CI non-applicable]
Blood Vessel Invasion	HR = 0.04 [95% CI non-applicable]
p53-status	HR = 1.59 [95% CI 0.33–7.55]
L1CAM-status	HR = 1.75 [95% CI 0.34–9.13]
Age at Surgery	HR = 0.98 [95% CI 0.86–1.12]
Indication for Surgery	HR = 7.47 [95% CI 1.47–37.96]
Complete Cytoreduction	HR = 0.41 [95% CI 0.11–1.57]
Tumor Grading	HR = 8.72 [95% CI 1.54–49.42]
Lymphatic Vessel Invasion	HR = 0.04 [95% CI non-applicable]
Blood Vessel Invasion	HR = 0.04 [95% CI non-applicable]
